# Polymeric coating doped with nanomaterials for functional impact on different substrates

**DOI:** 10.1038/s41598-023-50462-0

**Published:** 2024-01-05

**Authors:** Phool Shahzadi, Muhammad Amjad Majeed, Saba Ibrahim, Sabahat Asif, Razia Kalsoom, Irshad Hussain

**Affiliations:** 1 Glass and Ceramics Research Centre, PCSIR Labs. Complex, Lahore, Pakistan; 2 Department of Chemistry, Government College University, Lahore, Pakistan; 3 Department of Chemistry and Chemical Engineering, Syed Babar Ali School of Science and Engineering, Lahore University of Management Sciences (LUMS), DHA, Lahore, 54792 Pakistan; 4 PCSIR Laboratories, Islamabad, Pakistan

**Keywords:** Biogeochemistry, Chemistry

## Abstract

Microorganism contamination on substrate surfaces is arousing increasingly concern as a serious health issue. In this research work, antimicrobial water-based acrylic paint containing silver nanoparticles (Ag NPs) was prepared using the facile Ag+ in situ reduction process, in which AgNO_3_ and reducing agent sodium acrylate were refluxed with acrylic polymeric solution to obtain an antimicrobial and antifungal polymeric material for substrate coating. The Synthesized antimicrobial and antifungal water-based acrylic paint were characterized by different spectroscopic techniques. The FTIR and UV–Visible spectroscopic analyses were investigated to study the water-based acrylic paint structure as well as the significant impact of Ag NPs on the paint matrix. The UV–Visible and FTIR Spectra peak shows successful integration of Ag NPs within the polymer matrix without altering the core functional groups of the paint. The water based acrylic paint exhibited a strong antimicrobial activity, revealed substantial inhibition zones against all four strains of Gram negative represented by *Escherichia coli, Acinetobacter baumannii, Klebsiella pneumoniae* and Gram-positive represented by *Bacillus cereus*. The coated film on substrate also shows great inhibition zone which exhibit a strong antimicrobial activity. Moreover, water based acrylic paint also exhibited a great antifungal activity, revealed substantial zone of inhibition against the *Aspergillus niger, Aspergillus terreus* and *Rhizopus arrhizus* fungal strains. Also, the coated film showed the best adhesion at 50% and 80% solution of polymeric coating sample as compared to pure or very dilute sample coating. This innovative approach has the potential to revolutionize varies industries from healthcare to construction.

## Introduction

**Figure 1:**
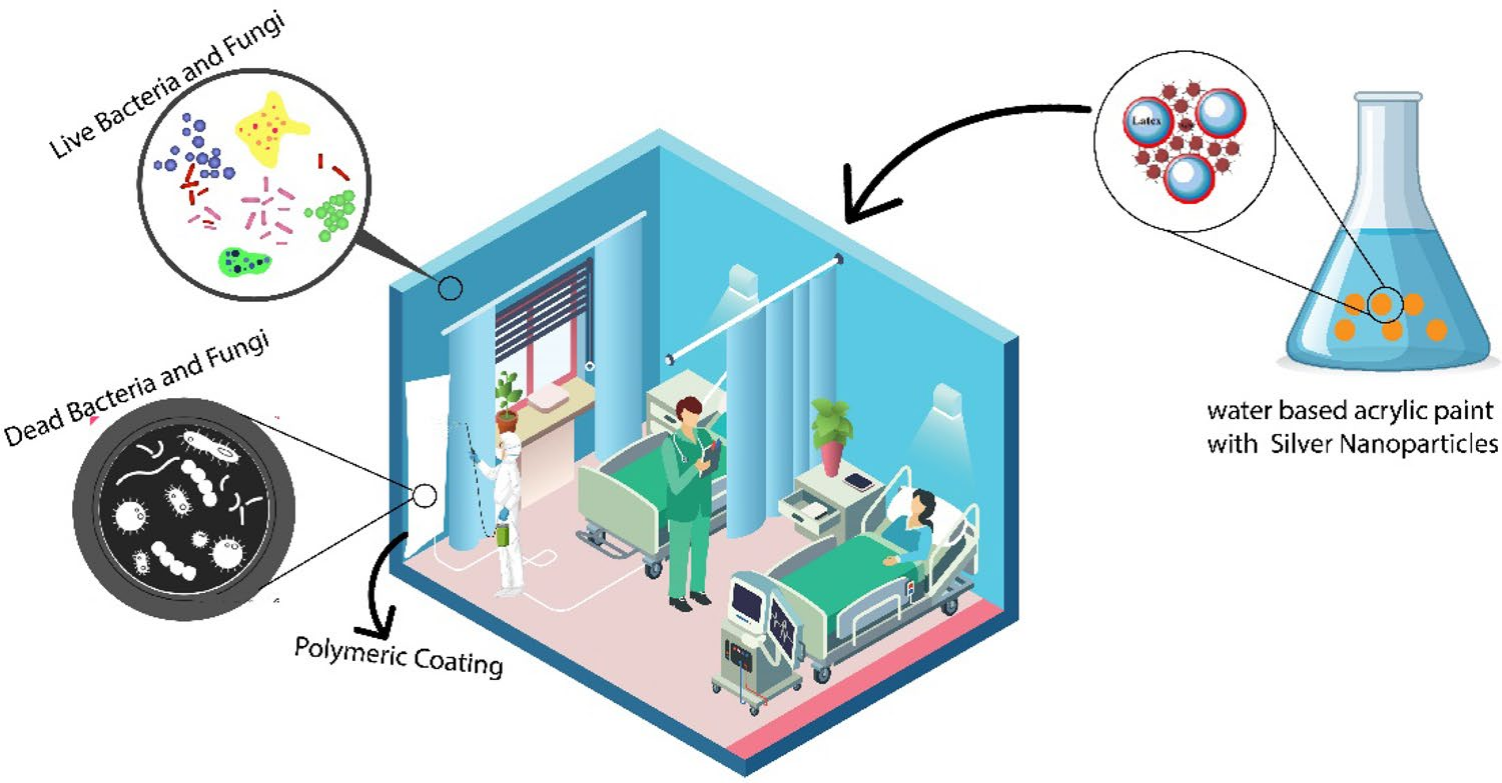
The graphical representation of Water based acrylic paint coating.

The resin has attracted much attention as a key component of the coating process. Coating is an integral stage in modifying the surface properties of materials^1,2^. Organic coatings have been used widely as protective and decorative materials for different kinds of substrate^3^. The viscosity of traditional solvent-based resins is reduced by organic volatile solvents^4^. As a result of a global focus on protecting the environment, laws and policies have been implemented that limit emissions of volatile organic compounds (VOCs)^5^. Due to these factors, traditional solvent-based coatings have been hindered which causes alternative coating technologies to be developed. The development of non-solvent coating systems, such as water-based and photo curable resin coatings, has been used in recent decades to reduce atmospheric pollutants^6^ (Fig. 1).

It is important to note that water-based paints are not only safe for the environment, but also comply with both European Biocidal Products Directive (BPD) and Environmental Protection Agency (EPA), which require that the VOC content of paints not exceed 350 g per liter of water^7^. In commercial production, acrylic latex paint is among the most common polymeric waterborne coatings, due to its ease of application and modification^8,9^. Acrylic resin provides cost-effective coatings that are highly resistant to weather, pollution, and both alkaline and acidic substances; thus, it is used widely in various industries as a cost-effective coating solution^10^. Additionally, these paints are low toxicity, highly resistant to atmospheric conditions, quick drying time at room temperature, and mainly compatible with a wide range of surfaces, including wood, mineral, and metal substrates^11^. A wide variety of microorganisms, whether created by humans or natural processes, pose an existential threat to the wellbeing of humans and many ecosystems around the world^12,13^. In order to prevent bacterial transmission from coatings to humans because bacteria easily transfer from coatings of any substrate to humans, so a proactive approach is particularly important^14^. A major challenge in hospitals, retirement homes, and kindergartens is the presence of resistant antibiotics bacteria in painted walls, like those associated with methicillin-resistant Staphylococcus aureus (MRSA). Due to the presence of cellulosic compounds such as thickeners in acrylic-based paints, algae, bacteria, and fungi can utilize the ingredients in paints for food and energy. Such environments must be controlled and prevented from spreading harmful microorganisms by addressing this issue^15^. Antimicrobial protection has been provided by a variety of organic and inorganic materials in the past but many of these materials have since been banned because of their harmful effects on humans and the environment. As a result, there is a need for further reductions in the use of such kinds of antimicrobial substances. Therefore, new environmentally-friendly alternatives to antimicrobial protection are becoming increasingly important^16^.

Through the incorporation of biocides as active agents, antimicrobial paints prevent or impede microbial colonization^17^. Nanoparticle-based antimicrobial compounds are more cost-effective and have a longer shelf life than traditional compounds. However, there are many factors that affect the toxicity of these nanomaterials against bacteria, including intrinsic properties, composition, surface modification, species of bacteria, and solvent composition etc.^18^. As far as antimicrobial properties are concerned, Ag NPs exhibit a greater level of efficacy than silver salts silver ions^19^. The bactericidal activity of Ag+ ions can be achieved at low concentrations ranging from 0.001 to 0.05 g/mL, but silver toxicity to human cells is much higher, so required much higher doses. The use of antimicrobial coatings in healthcare facilities and public places has become increasingly important in controlling infections^20^. While these coatings do not completely eliminate infection transmission, they have proven to reduce it by a considerable amount^21^. Nishimura et al. using sodium acrylate as a dual reducing and capping agent, various analytical techniques have been used to investigate the formation mechanism of Ag NPs. They Successfully Synthesized the Ag NPs through sodium acrylate^22^. Bin Feng et al. developed an eco-friendly Nano-silver hydrosol from soy protein isolate to meet the increased demand for antibacterial products during the COVID-19 pandemic. They created a polyacrylate paint based Nano silver antibacterial wood coating using this hydrosol, which demonstrated excellent antibacterial activities against both gram-positive and gram-negative bacteria^23^.Similarly, Fatemeh Farsinia et al. investigated the antibacterial properties of Ag-RGO nanocomposite in water-based acrylic paints. The hybrid material showed strong antibacterial properties against *Escherichia coli* and *Staphylococcus aureus*. Increasing the Ag-RGO concentration improved inhibition efficiency, making it a promising antibacterial agent^24^. But the purpose of this work was to develop a new waterborne acrylic paint that would exhibit both chemical resistance properties and biocidal efficiency in the resulting paint films. A one-component water-based acrylic polymeric dispersion paint was synthesized, with the in-situ synthesis of silver nanoparticles (PSK2) through sodium acrylate. A reference system was also formulated with the same composition, but without nanoparticles (PSK1). For coating also facile method of dip coating used which is economically cheap method. Our product not only shows the antibacterial activity, but it also shows antifungal activity which makes it unique from other works. The efficacy of the resulting paint films was evaluated in terms of their biocidal effect, with a comparison between silver nanoparticles based polymeric film and transparent polymeric films.

## Experimental section

### Materials

Nutrient browth (CM0001) was purchased from OXOID LTD. Basingstoke, Hampshire, England, and agar, Bacteriological (CAS: 9002-18-0) purchased from bio plus Chemicals. Dextrose, Ethanol, Silver nitrate(AgNO_3_), Sodium acrylate, Butyl acrylate (BA), methyl methacrylate (MMA) and methacrylic acid (MAA), Potassium persulfate (KPS, Sigma-Aldrich), Titanium oxide were purchased from Sigma-Aldrich, ammonia water (E.Merk), Acrylic Dispersant,Natrosol 250-HR,NP-9(sigma-Aldrich) Texanol were purchased from Dow Chemical Company (Thailand), Mergal, propylene glycol,Antifoam,brighty 425 mesh, P-820, China Clay, Talcum powder, Laponite Gel were purchased from Guangdong Weng Jiang Chemical Reagent Co., Ltd from China and 2-(methacryloyloxy)ethyl acetoacetate (AAEM) were purchased from Shanghai, China.

### Method

#### Synthesis of water-based acrylate paint

The paint consists of two main components: the binder and the mill base. The binder includes various ingredients such as pigment, extender, surfactant, thickener, and more. The formulation for a water-based acrylic emulsion is provided in Table 1. To prepare the acrylic resin, reactions were conducted in a nitrogen atmosphere using a four-necked flask with a condenser pipe and magnetic stirrer. A mixture of 100 g H_2_O, 1.5 g NP-9 emulsifiers, and 0.05 g KPS was added to the flask and refluxed at 82 °C. The remaining monomers (12% Methyl methacrylate, 16% butyl acrylate, and 2-(methacryloyloxy) ethyl acetoacetate) were placed in one beaker, while the rest of KPS and H_2_O were in another. Both mixtures were gradually added to the flask over 3–4 h, followed by an additional 2 h reaction. After cooling and pH adjustment to 7, the emulsion was discharged. For the mill base, 20 ml distilled water and 0.50 g Natrosol 250-HR were combined in a three-necked bottle with a stirrer. After stirring and dissolving, 0.30 g dispersant was added, and stirring continued for 15 min. Subsequently, 1 g propylene glycol butyl ether was added, and high-speed stirring took place at 800 r/min, followed by a reduction to 300r/min for ongoing slow stirring. Then, 6 g talcum powder, 18 g titanium dioxide powder, and other additives (China clay, P-820, Mergal) as specified in the table were added. Additionally, 32.55 g of freshly prepared acrylic emulsion, along with remaining water, was added. After 0.5 h of stirring, 0.10 g of defoaming agent was introduced, and the pH was adjusted to 7.0 using ammonia water. Stirring for 20 min was performed before discharging to obtain the final product.

**Table 1:** A standard formulation for water based acrylic Emulsion paint.

Raw material	%age	Mol.Wieght (g/mole)	Molecular formula	Role
Water	20.00	18.00	H_2_O	Solvent
Acrylic Dispersant	00.30	–	–	improve dispersion stability
Natrosol 250-HR	00.50	736.7	C_29_H_52_O_21_	Thickener
NP-9	00.30	616.8235	C_33_H_60_O_10_	Used as an emulsifier, wetting agent, dispersant
Mergal	00.15	93.51	C_2_H_4_ClNO	Micro biocide
Propylene Glycol	01.00	76.09	C_3_H_8_O_2_	De-icing
Ammonia	00.10	17.031	NH_3_	pH regulator
Antifoam	00.10			Defoamer
TiO_2_-595	18.00	79.866	TiO_2_	White pigment
Brighty 425 mesh	11.00	_–_	–	
P-820	05.00	119.06	C_10_H_15_NO	Filler
China Clay	05.00	258.16	Al_2_O_2_∙2SiO_2_∙2H_2_O	Extender
Talc	06.00	379.27	Mg_3_Si_4_O_10_(OH)_2_	Increases coverage and weather resistance
Acrylic Resin	22.00	500–10,000	[CH_2_=C·(CH_3_) COOH]	
Texanol	02.00	216.3172	C_12_H_24_O_3_	Highest level of film integrity at low levels of coalescent
Laponite Gel	05.00	2286.9	NaO. 7Si_8_Mg_5_. 5LiO. 3O20(OH)_4_	To remove old water-soluble adhesives
Ammonia	00.10	17.031	NH_3_	pH regulator
Antifoam	00.10			Defoamer
Water	03.35	18.00	H_2_O	Solvent
Total	100.00			

#### In situ synthesis of silver nanoparticles

The silver nanoparticles were grown inside the polymeric solution. For this 5% by weight 50 mL aqueous solution of polymer was prepared and stirred for about 30 min followed by the addition of AgNO_3_ (25.48 mg). Reaction mixture was refluxed for 5–10 min under an argon atmosphere, and a warm (50–60 °C) aqueous solution of sodium acrylate (25 mL, 160 mM) was added quickly. The mixture was further refluxed for 40 min, which resulted in silver nanoparticles coated by polymer. The average size of silver nanoparticles was around 19.7 nm.

#### Characterization and testing

The water-based acrylic antimicrobial paint was analyzed by Agilent Technologies, Cary 60 UV–visible spectrophotometer. As the blank control, distilled water was used. Fourier transform infrared (FTIR) spectra of samples were obtained on an IR Prestige-21(SHIMADZU) spectrometer in the range of 4000–400 cm^−1^. The surface morphologies of the water-based acrylate doped with silver nanoparticles and composite films were assessed by a scanning electron microscope (SEM).

##### Antibacterial activity

*Escherichia coli, Acinetobacter baumannii, Klebsiella pneumoniae* were selected as the representative gram-negative bacteria, *Bacillus cereus*. was selected as the representative of gram-positive bacteria, and to evaluate the antibacterial activity of the water-based antimicrobial acrylic paint.

##### Biological required materials and the collection of bacteria for antimicrobial activity

Nutrient browth (CM0001) was purchased from OXOID LTD. Basingstoke, Hampshire, England, and agar, Bacteriological (CAS: 9002-18-0) purchased from bio plus Chemicals. Bacterial strains which include three gram-negative bacteria that is Escherichia coli, Acinetobacter baumannii, Klebsiella pneumoniae and one-gram positive bacteria *Bacillus cereus* were collected from the collection of bacteria of different patients from the microbiology lab of Mayo Hospital, Lahore.

##### Zone of inhibition determination through well-diffusion method

The well diffusion method was employed to assess the susceptibility of the bacterial strain to the newly synthesized water-based acrylic antimicrobial paint. Various bacterial strains were sub-cultured using the Muller Hinton agar streak technique, and then incubated at 36–37 °C for 22–24 h. Fresh agar media was prepared by heating and stirring distilled water, followed by autoclaving at 121 °C and 15 pounds of pressure for 15 min. At 40–50 °C cooling, the agar was poured into sterile petri dishes and allowed to solidify. The agar plates were divided into three sections: PSK1, PSK2, and antibiotic. Sterilized cotton swabs were immersed in standardized bacterial suspensions and used to streak the agar surface. Well-defined wells were created using sterile pipette tips and filled with samples: PSK1, PSK2, and a reference antibiotic. Incubation was conducted at the optimal growth temperature of 35 to 37 °C for 24 h. The diameter of the clear inhibition zones was measured in millimeters. This process was repeated three times, yielding consistent results in each iteration^25^.

##### Coated substrate antibacterial activity

The sterile stainless-steel substrates (1 cm × 1 cm) were coated with PSK1 and PSK2. Similarly Fresh bacteria cultures were inoculated onto nutrient broth and incubated at 37 °C for 24 h. The sterile plates were poured with Müller-Hinton agar and solidified. Using inoculated agar, the stainless-steel substrate was impregnated. After inoculation, plates were incubated for 22 to 24 h at 37 °C. The inhibition zones were observed to determine the antibacterial activity of the coating.

##### Antifungal activity

*Aspergillus niger, Aspergillus terreus and Rhizopus arrhizus* were used as a fungal strain to evaluate the antifungal properties of the water based acrylic paint doped with silver Nanoparticles.

##### Fungal culture collection, culture media and inoculums preparation

Different Fungal Strain Culture were collected from the Biotechnology department laboratory from Government college university, Lahore. Potato dextrose agar was prepared from potato extract (200 g potato in 100 ml distilled water). In potato extract 4 g dextrose and 4 g agar and 1–2 drop antibiotic to stop bacterial growth dissolved with continuous stirring and heating to prepare 200 ml potato dextrose agar (PDA) and autoclave it at 121 °C temperature for 20 min. Fungal strains were cultured on potato dextrose agar (bio plus Chemicals). A loop full of all the fungus cultures were inoculated with freshly prepared potato dextrose Agar (PDA) and incubated at room temperature for 72 h.

##### Measuring zone of inhibition for antifungal activity

Autoclaved Potato dextrose agar (PDA) pour into sterile plastic petri dishes and provide some time to agar medium cool and solidify. The fresh agar plates were divided into three portions PSK1, PSK2 and antifungal reference. The PDA plates were inoculated with the fungal organism using a sterile swab to spread the inoculum evenly over the surface of the agar. Sterile pipette tips were used to make wells in the agar plates to fill the samples. The test compounds were added to the wells using a sterile pipette. The sterile agar plates were incubated at 31 °C for 72 h. The activity of the samples was determined by measuring the zone of inhibition diameters. For each fungal strain, controls were maintained where pure PSK1 paint without silver nanoparticles were used^26^.

### Pretreatment of substrates and coating

Before coating, activating the substrate’s surface can improve adhesion and promote a stronger bond between the substrate and the coating. To remove dirt, dust, or grease from the substrates, thoroughly clean it with soap and water. Clean the substrate by rinsing with deionized water and wiping it dry with a lint-free cloth. To improve adhesion, glass and metal substrate are treated with a surface conditioner. For improved adhesion and to prevent tannin bleeding, treat the wood substrate with a wood sealer or primer. The wood coatings were prepared by brush coating an emulsion on a wooden board. The wooden board was then subjected to an oven at 80 °C for 30 s. Water was then removed from the wood coating, and then it was irradiated with 395 nm UV lights for 10–15 s. The dip coating was used by making 80% and 50% solution of water based acrylic antibiotic paint, stainless steel and mild steels Substrates were immersed in a solution or suspension of a material to be coated. At a controlled speed, the substrate is withdrawn, allowing a thin film to form. After the coating is applied, the substrate is dried and cured to achieve the desired properties. The paint film was evaluated 10 days after it had been completely dried.

## Result and discussion

### Scanning electron microscopy (SEM)

**Figure 2:**
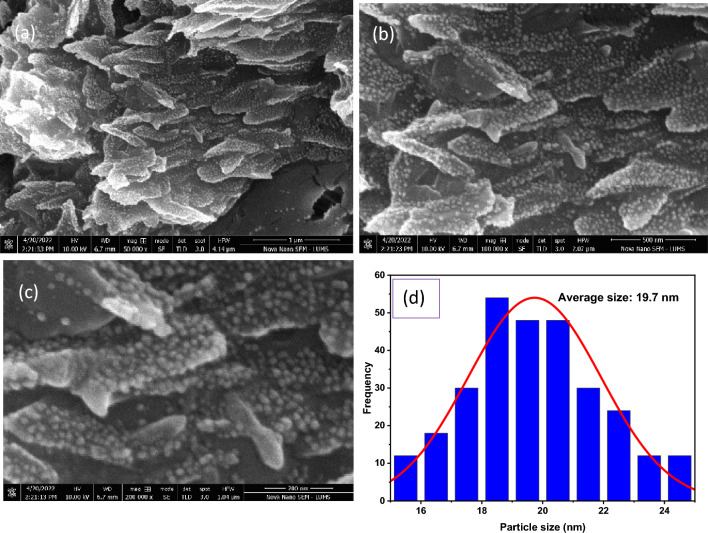
SEM imaging of water based acrylic paint with Ag NPs (**a**) mag 50,000 X (**b**) mag 100,000 X (**c**) mag 200,000 X (**d**) average particle size of Ag NPs.

Through SEM imaging, Ag NPs surface morphologies were characterized in water-based acrylic films. SEM images of water-based acrylic paint film containing silver nanoparticles are shown in Fig. 2. Ag NPs can be seen in the SEM images as white spots. The results indicate that nano-silver was dispersed in acrylic paint without agglomerating on a large scale. The average particle size has been measured which is 19.7 nm, and the size distribution graph is depicted in Fig. 2d.

### FT-IR analysis

**Figure 3:**
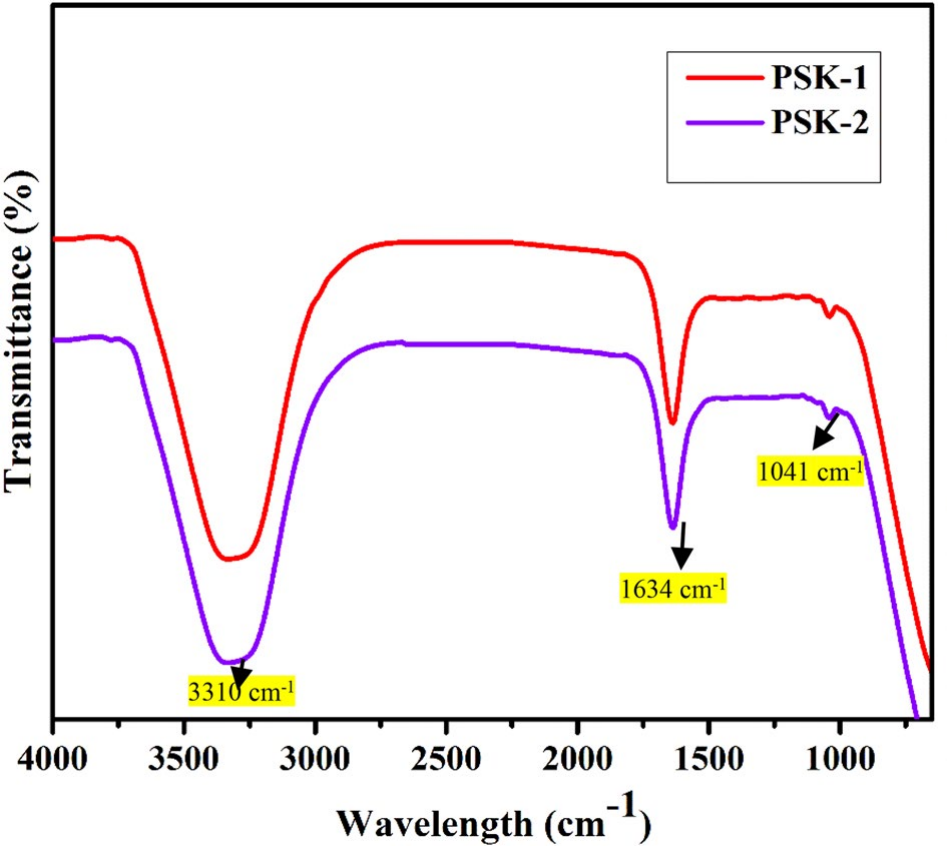
FTIR spectra of water-based acrylate PSK1 and doped with silver nanoparticle PSK2.

FTIR spectroscopy characterization can identify the main functional groups in the molecular structure and studying the influence of Ag NPs on core functional groups. The results are shown in Fig. 3. The infrared (IR) spectrum of the compound under investigation exhibited characteristic absorption bands at 3310 cm^−1^, 1634 cm^−1^, and 1041 cm^−1^. The strong and broad stretching vibration peaks observed at 3310 cm^−1^ suggests the presence of an O–H bond, indicating the possible presence of an alcohol (–OH) residual from monomers or absorbed moisture. The absorption band at 1634 cm^−1^ indicates the presence of a C=O bond stretching band, which is commonly observed as a carbonyl group, common in acrylic polymers due to ester linkages. Additionally, the absorption peak at 1041 cm^−1^ suggests the presence of a C–O bond stretching band, commonly found in ethers, or esters^27–29^.These findings suggest the presence of an alcohol functional group (–OH), a carbonyl group (C=O), and a compound containing a C–O bond^30^. These Groups suggest that both PSK1 and PSK2 are not affected by silver nanoparticles and these nanoparticles caped by the polymeric compound. Although show strong antibacterial activity against bacterial strains due to silver nanoparticles which is evaluated in antimicrobial susceptibility test.

### UV–visible spectroscopy

**Figure 4:**
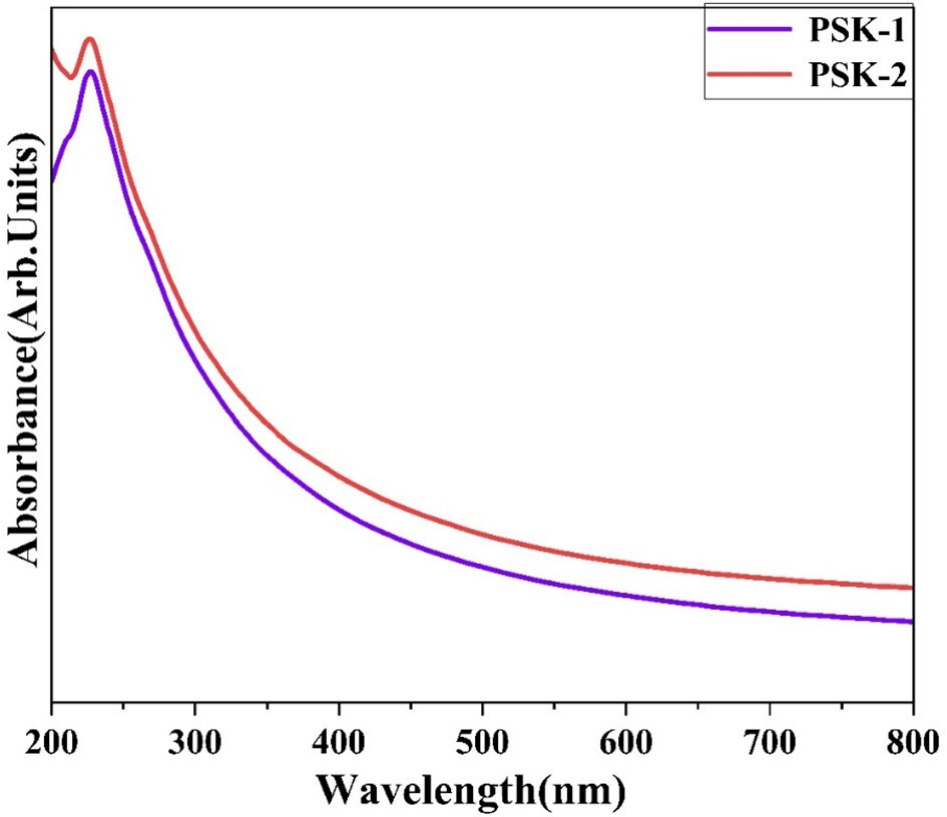
UV–Visible spectra of water-based acrylate PSK1 and doped with silver nanoparticle PSK2.

The absorption characteristics of paint were investigated using UV–visible spectroscopy within the wavelength range of 200–800 nm. The aim was to examine the influence of silver nanoparticles on the absorption properties. The observed peak at 224 nm was attributed to water-based acrylic paint^31^ as shown in Fig. 4.The introduction of silver nanoparticles did not result in any noticeable shift in the peak position. This suggests that the interaction between the silver nanoparticles and the acrylate paint matrix may not have a significant impact on the electronic environment responsible for the observed absorption band. It is worth noting that the lack of discernible effects could be attributed to the exceptionally low concentration of silver nanoparticles utilized.

### Antimicrobial activity

**Figure 5:**
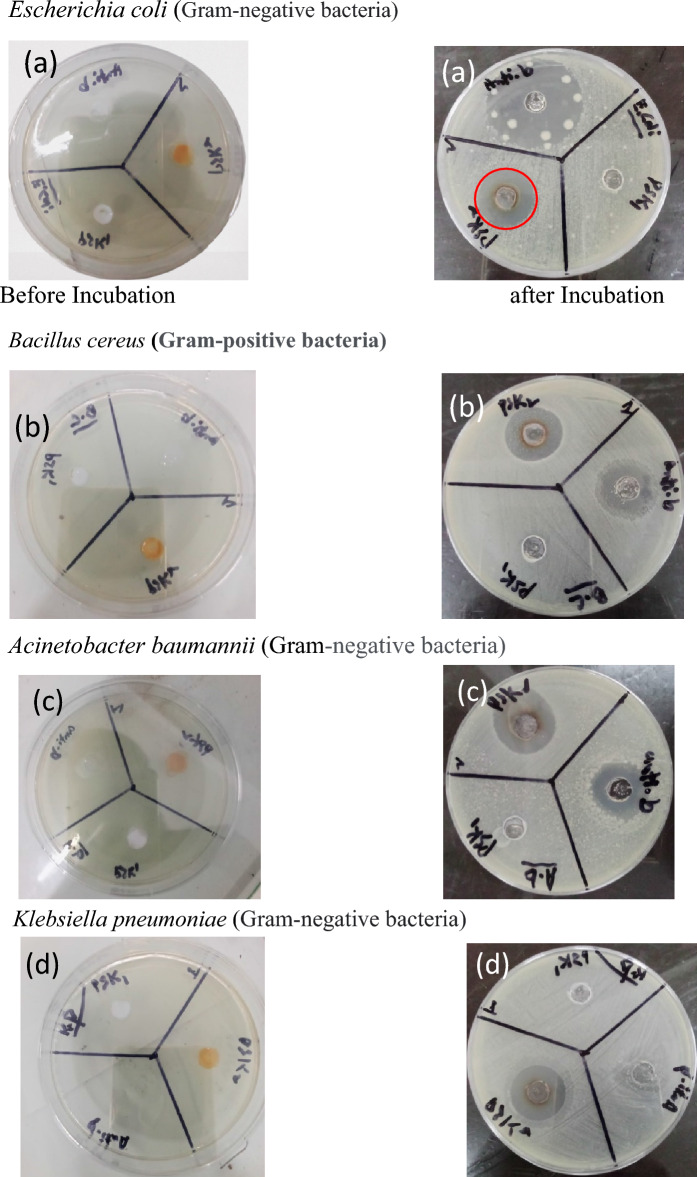
Results of the antibacterial activity of PSK1 and PSK2 before and after incubation (**a**), *Escherichia coli* (**b**), *Bacillus cereus* (**c**), *Acinetobacter baumannii* and (**d**) *Klebsiella pneumoniae*.

**Figure 6:**
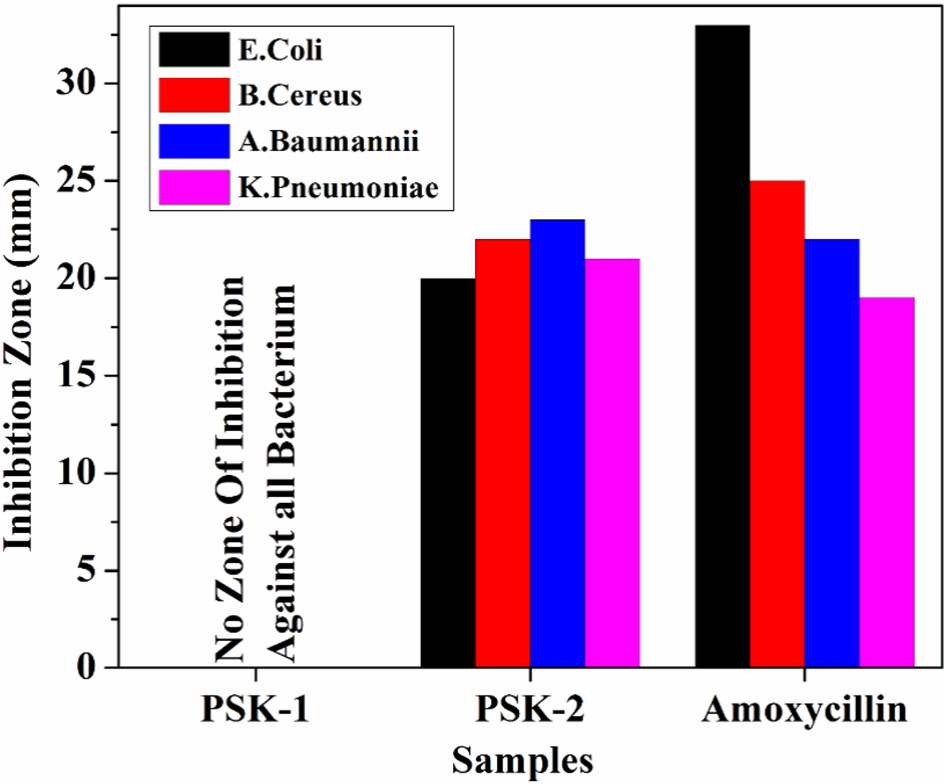
Diameter of Inhibition Zone (mm) of PSK1, PSK2 and reference antibiotic Amoxicillin against different Bacterium.

The well diffusion method was used to evaluate the antimicrobial activity of the water-based acrylate paint in which silver nanoparticles were doped. Pure water based acrylic paint PSK1 and doped with silver nanoparticle PSK2 antimicrobial activity were evaluated through the reference antibiotic material. The prepared water based acrylic paint doped with silver nanoparticle film had excellent resistance to *Escherichia coli, Acinetobacter baumannii, Klebsiella pneumoniae* (gram negative bacteria) *Bacillus cereus* (gram-positive bacteria). The silver nanoparticles in paint caused structural damage to bacteria and cell death because of its interaction with the cell membrane. By blocking ribosomal subunit proteins, inhibiting ATP production processes, and affecting DNA replication, it destroyed respiratory chain binding enzymes, inhibited ribosomal subunit proteins^24^. The results of antimicrobial activity are shown in Table 2 and illustrated by Figs. 5, 6.

**Table 2:** Results of antibacterial activity.

Organism	PSK-1	PSK-2 (mm)	ANTIBIOTIC (Amoxicillin) (mm)
*Escherichia coli*	No zone of Inhibition	20 ± 1	33 ± 1
*Bacillus cereus*	No zone of Inhibition	22 ± 1	25 ± 1
*Acinetobacter Baumannii*	No zone of Inhibition	23 ± 1	22 ± 1
*Klebsiella pneumoniae*	No zone of Inhibition	21 ± 1	19 ± 1

### Coated substrate antimicrobial testing

**Figure 7:**
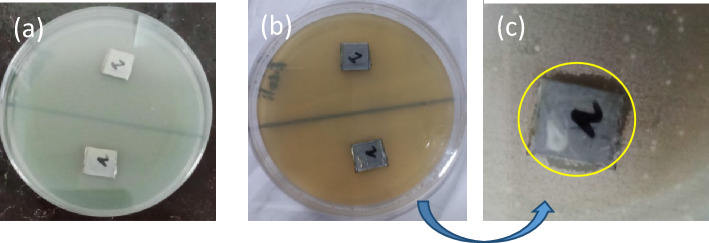
Coated stainless steel Substrate with PSK1 (as mentioned 1) and with PSK2 (as mentioned 2) (**a**) Before incubation (**b**) After incubate at 37 °C for 24 h (c) Zoomed, antibacterial activity against *Escherichia coli.*

*Escherichia coli* and *Acinetobacter baumannii* as Gram negative species were subjected to the PSK1 and modified with silver nanoparticles PSK2 coating to determine the antibacterial effect of the coatings. Coatings were casted on Stainless steel. As shown in Fig. 7, the PSK1 coatings (mentioned as 1 in fig.) had no zone of inhibition but PSK2 coating on substrate (mentioned as 2 in fig.) show zone of inhibition PSK2 coating show more than 60% antibacterial activity against *Escherichia coli* which is great result. As silver ions leached from the PSK2 coating, they could migrate to the agar surface next to the disks. As a result, Gram negative bacteria would not be able to grow near the coating.

### Antifungal activity

**Figure 8:**
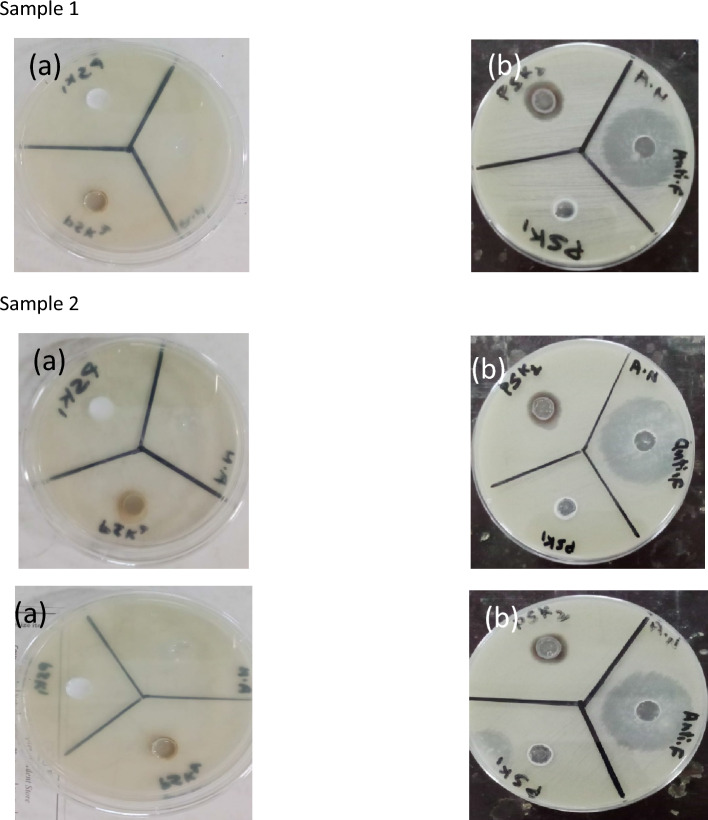
Results of the antifungal activity of PSK1 and PSK2 against *Aspergillus niger* (A.N) (**a**) before incubation and (**b**) after incubation of 72 h.

**Figure 9:**
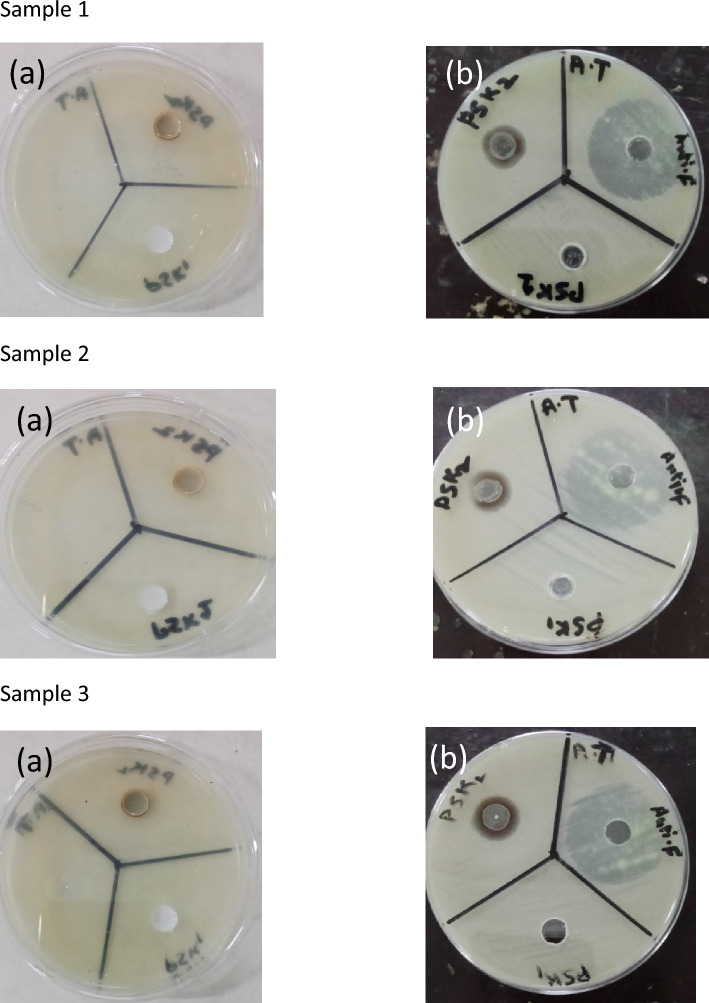
Results of the antifungal activity of PSK1 and PSK2 against *Aspergillus terreus* (A.T) (**a**) before incubation and (**b**) after incubation of 72 h.

**Figure 10:**
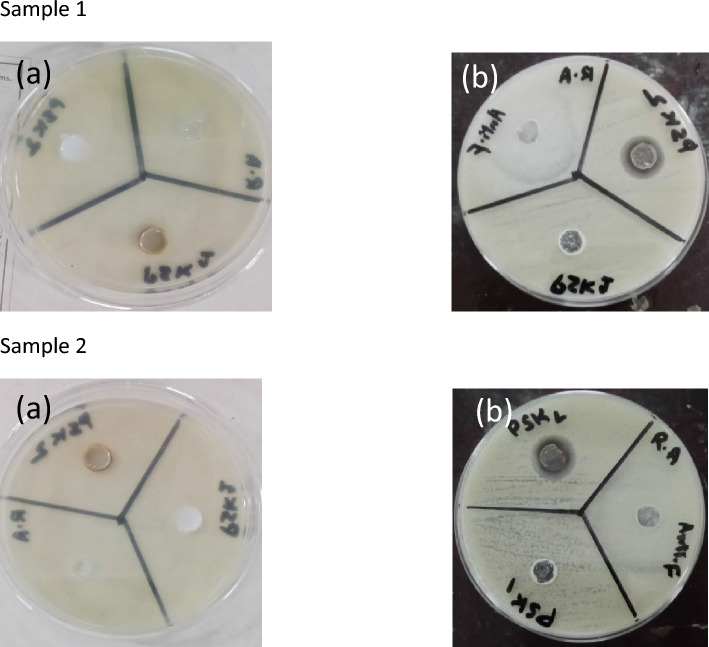
Results of the antifungal activity of PSK1 and PSK2 against *Rhizopus arrhizus* (R.A) (**a**) Before incubation and (**b**) after incubation of 72 h.

**Figure 11:**
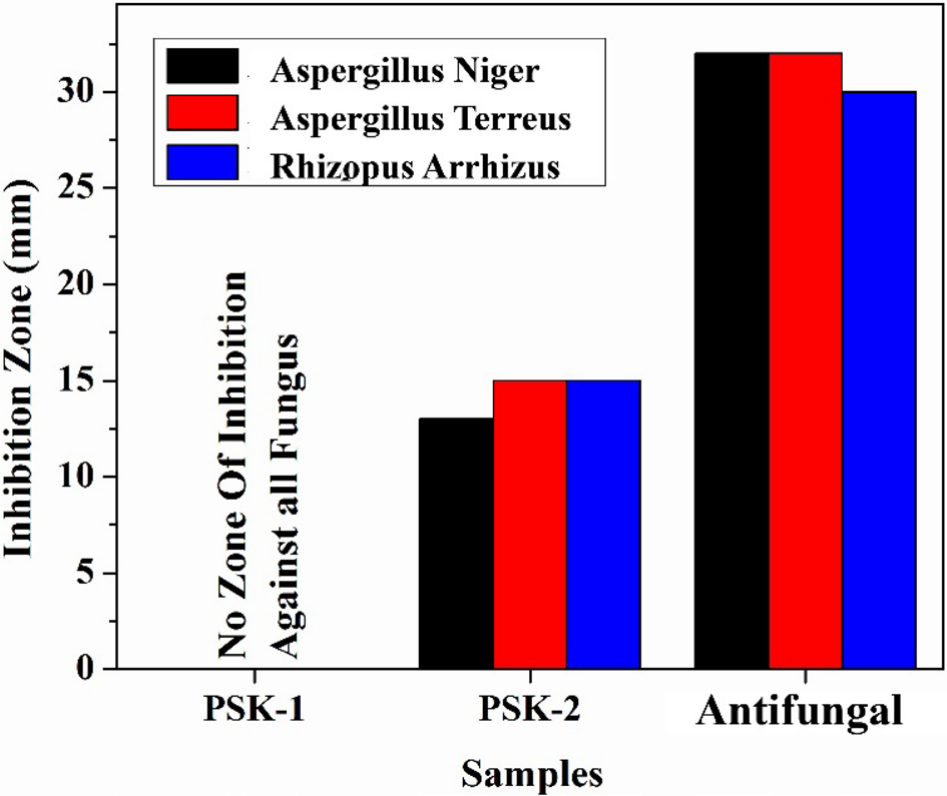
Diameter of Inhibition Zone (mm) of PSK1, PSK2 and reference antifungal amphotericin against different fungal strains.

For antifungal activity well diffusion method was also used to evaluate the antifungal activity of the water-based acrylate paint in which silver nanoparticles were grown. Pure water based acrylic paint PSK1 and doped with silver nanoparticle PSK2 antifungal activity were evaluated through reference antifungal amphotericin medicine. The prepared water based acrylic paint doped with silver nanoparticle film had excellent resistance to *Aspergillus niger*, *Aspergillus terreus and Rhizopus arrhizus*. The results of antifungal activity show in Tables 3, 4, and 5 and illustrated by Figs. 8, 9, 10, and 11.

**Table 3:** Results of Antifungal Activity of samples against *Aspergillus niger* (A.N).

Sample no	PSK 1	PSK 2 (mm)	Antifungal (mm)
Sample 1	No zone of Inhibition	13	32
Sample 2	No zone of Inhibition	13	32
Sample 3	No zone of Inhibition	14	31

**Table 4:** Results of antifungal activity of samples against *Aspergillus terreus* (A.T).

Sample no	PSK 1	PSK 2 (mm)	Antifungal (mm)
Sample 1	No zone of inhibition	15	32
Sample 2	No zone of inhibition	15	33
Sample 3	No zone of inhibition	15	33

**Table 5:** Results of antifungal activity of samples against *Rhizopus arrhizus* (R.A).

Sample no	PSK 1	PSK 2 (mm)	Antifungal
Sample 1	No zone of inhibition	15	30
Sample 2	No zone of inhibition	15	Not clear

**Figure 12:**
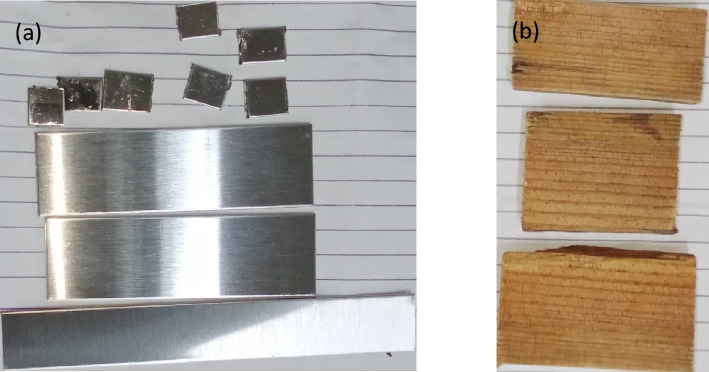
after surface treatment substrate before coating (**a**) stainless steel and mild steel (**b**) wood.

**Figure 13:**
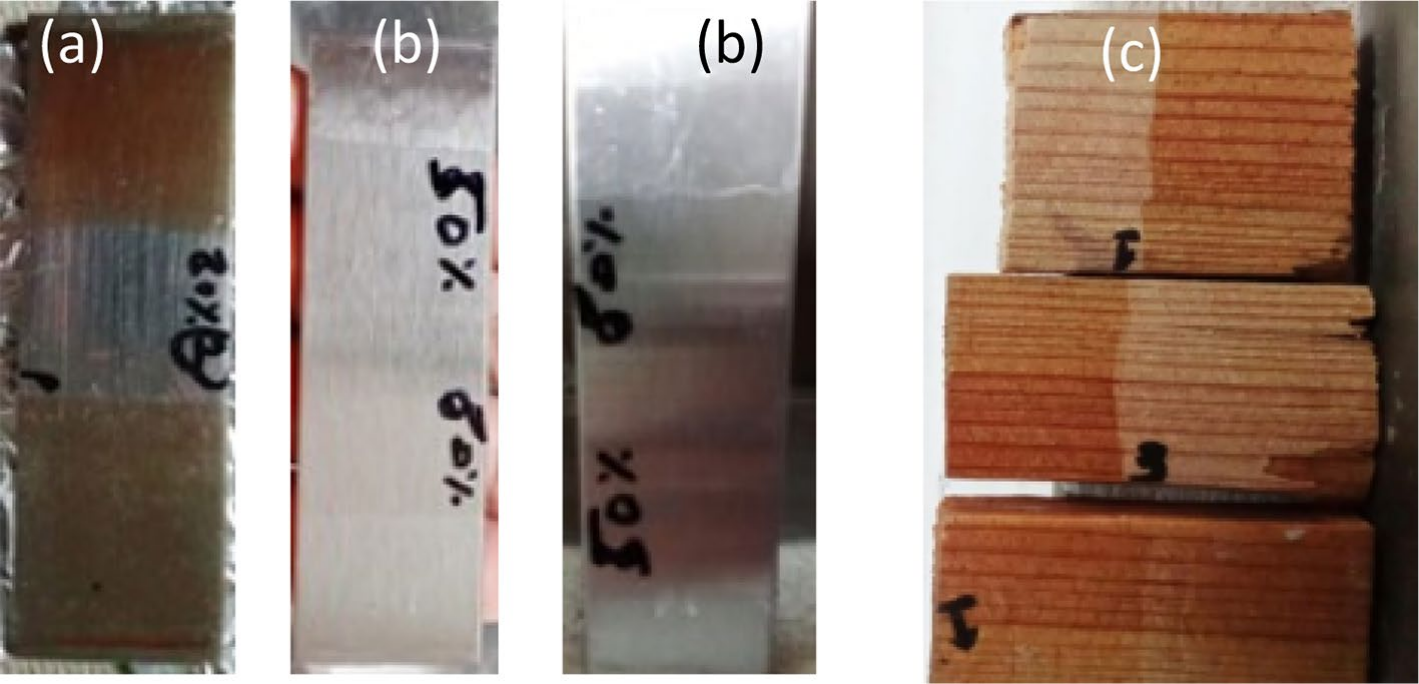
After coating of substrate in pure, 80% and 50% (**a**) Mild steel (**b**) stainless steel (**c**) wood.

The results of the adhesion tests performed after 10 days of complete drying revealed a substantial improvement in coating adhesion for all substrates. As Shown in Fig. 12 the surface activation process significantly increased the bond strength between the substrate and the coating. After 10 days of complete drying of substrate’s coating, observed that 80% and 50% solution of water-based acrylate coating provide good adhesion and clear coating as compared to pure sample coating as shown in Fig. 13.

## Conclusion

The antibacterial and antifungal activities of PSK1 and PSK2 were examined against different bacterial and fungal strains. This study was designed to address the challenges posed by microbial colonization of coatings and surfaces, particularly those found in healthcare facilities and public spaces. The study addresses the challenges associated with microbial colonization on surfaces and coatings, especially in healthcare facilities and public spaces. The incorporation of silver nanoparticles with polymeric matrix at the nanoscale has demonstrated promising results in combating both gram-negative and gram-positive bacteria, as well as different fungal strains. The results UV–Vis spectroscopy, FT-IR analysis, and SEM imaging suggest that silver nanoparticles were successfully integrated within the polymer matrix without altering its core functional groups. Several microorganisms were significantly inhibited by the water-based acrylate paint with silver nanoparticles, indicating its potential as an antimicrobial coating. In the context of growing concerns about microbial transmission and infection control, research indicates that environmentally friendly antimicrobial alternatives may be more effective than traditional antimicrobials.

Overall, water-based acrylic paints with silver nanoparticles have enormous potential as multifunctional coating materials that are antimicrobial and antifungal. Healthcare, construction, and countless other industries can benefit from this innovative approach by maintaining hygienic and safe environments. Further study in this field could lead to widespread adoption of these coatings, resulting in improved public health and sustainability of the environment.

## Data Availability

The datasets used and analyzed during the current study are available from the corresponding author on reasonable request.
